# Circular RNAs in Embryogenesis and Cell Differentiation With a Focus on Cancer Development

**DOI:** 10.3389/fcell.2020.00389

**Published:** 2020-05-27

**Authors:** Silvia Di Agostino, Anna Riccioli, Paola De Cesaris, Giulia Fontemaggi, Giovanni Blandino, Antonio Filippini, Francesco Fazi

**Affiliations:** ^1^Oncogenomic and Epigenetic Unit, Department of Diagnostic Research and Technological Innovation, IRCCS Regina Elena National Cancer Institute, Rome, Italy; ^2^Department of Anatomical, Histological, Forensic & Orthopedic Sciences, Section of Histology & Medical Embryology, Sapienza University of Rome, Rome, Italy; ^3^Department of Biotechnological and Applied Clinical Sciences, University of L’Aquila, L’Aquila, Italy; ^4^Laboratory Affiliated to Istituto Pasteur Italia-Fondazione Cenci Bolognetti, Sapienza Università di Roma, Rome, Italy

**Keywords:** circRNA, embryogenesis, development, stemness, cancer

## Abstract

In the recent years thousands of non-coding RNAs have been identified, also thanks to highthroughput sequencing technologies. Among them, circular RNAs (circRNAs) are a well-represented class characterized by the high sequence conservation and cell type specific expression in eukaryotes. They are covalently closed loops formed through back-splicing. Recently, circRNAs were shown to regulate a variety of cellular processes functioning as miRNA sponges, RBP binding molecules, transcriptional regulators, scaffold for protein translation, as well as immune regulators. A growing number of studies are showing that deregulated expression of circRNAs plays important and decisive actions during the development of several human diseases, including cancer. The research on their biogenesis and on the various molecular mechanisms in which they are involved is going very fast, however, there are still few studies that address their involvement in embryogenesis and eukaryotic development. This review has the intent to describe the most recent progress in the study of the biogenesis and molecular activities of circRNAs providing insightful information in the field of embryogenesis and cell differentiation. In addition, we describe the latest research on circRNAs as novel promising biomarkers in diverse types of tumors.

## Introduction

With the advent of next-generation sequencing, the list of diverse non-coding RNA species with functional capacity expressed in eukaryotic cells has grown very rapidly and some computational algorithms emerged to predict circRNAs, which were most commonly found at back-splicing junctions (Veneziano et al., 2016; Gao and Zhao, 2018).

**FIGURE 1:**
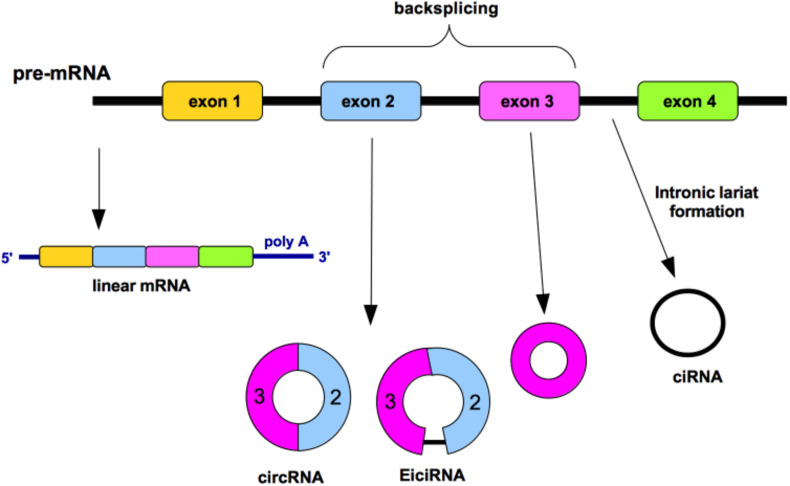
circRNA generation by “backsplicing” mechanism. Diverse circular RNAs (circRNAs) can be formed from a single gene. The non-canonical splicing process named “backsplicing” is the responsible of the circRNA formation. A downstream splice donor is joined to an upstream splice acceptor generating diverse isoforms. Such circRNAs can consist of one or more exons and can even contain unspliced intronic sequences. Circularized intron RNAs are not produced by backsplicing, rather by an inefficient debranching. Colored bars, exons; black lines, introns.

Circular RNA (circRNA) is a type of single-stranded RNA usually formed by alternative splicing of pre-mRNA where the 5′ upstream splice acceptor is joined to 3′ downstream splice donor in a process named “backsplicing” (Figure 1). This event forms covalently closed continuous loops without polyadenylated tails and, as result, circRNAs are insensitive to the majority of exoribonucleases (Kristensen et al., 2019). CircRNAs are classified into three categories: exonic circRNAs (ecircRNAs; with one or more exons) that represent 85% of all circRNAs, exonic-intronic circRNA (EicirRNA) and circularized intronic RNA (ciRNA) (Kristensen et al., 2019; Figure 1). A competitive relationship may exist between the linear RNA splicing and the back-splicing events. The two introns flanking the circularized exons, which have been found to be enriched in Alu repeats, usually increase the efficiency of circularization (Zhang et al., 2014).

Initially, circRNAs were occasionally discovered by RT-PCR amplification and sequencing (Nigro et al., 1991; Cocquerelle et al., 1992). Only 20 years later, to find genomic rearrangements in cancers, the expression of circRNAs was discovered through RNA-seq in human pediatric acute lymphoblastic leukemia (Salzman et al., 2012). The authors showed that this phenomenon could be extended to leukocytes from healthy adults as well as to several other cancer and non-cancer cell lines and to mouse brain (Salzman et al., 2012).

The most important features of circRNAs can be summarized as follows: (a) circRNAs are abundant forms of non-coding RNAs that are expressed from thousands of human genes, sometimes even at higher level than their cognate linear isoforms (Salzman et al., 2012; Zhang et al., 2014; Kristensen et al., 2019); (b) circRNAs exhibit cell type-specific expression (Chen, 2016); and (c) circRNAs present a high rate of conservation between mouse and human and are quite stable molecules, with half-lives exceeding 48 h (Jeck et al., 2013).

**FIGURE 2:**
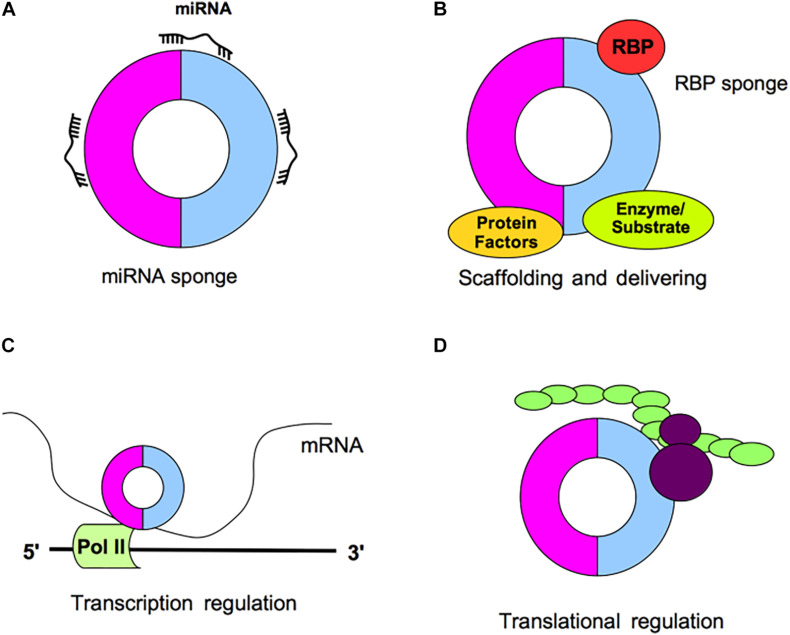
Schematic representation of circRNA functions. **(A)** CircRNAs may act as miRNA sponges by competing for miRNA binding sites, decreasing the miRNA activity on the mRNA targets. **(B)** CircRNAs may act as protein sponges, by binding RNA-binding proteins (RBPs) or acting as platform for enzymatic reactions or acting as a protein platform. **(C)** CircRNAs may regulate the transcription. **(D)** CircRNAs may interact with ribosomes and affect protein translation.

Circular RNAs could have multiple functions within the cell acting for example as miRNA sponges, by competing for miRNA binding sites and thus decreasing the miRNA activity on the target mRNA, or acting as protein sponges; moreover, circRNA can interact with RNA-binding proteins (RBPs), act as platform for enzimatic reactions or act as a protein platform (Figure 2); finally, circRNAs may regulate the transcription and may interact with ribosomes thus affecting protein translation (Kristensen et al., 2019; Figure 2).

## CircRNAs in Embryogenesis

The embryo development begins when the genetic transcription of zygote is activated. Different from the intensely studied mRNAs, circRNAs are still in the opening of this research field. By means of deep sequencing and bioinformatics technologies, the set of circRNA expressed in human pre-implantation embryos have been reported (Fan et al., 2015; Dang et al., 2016). Embryonic stem cells derived from the pluripotent inner cell mass of the blastocyst are clonogenic and have the ability for unlimited self- renewal and pluripotency, leading to all cell types in the human body tissues. That ncRNAs play an important role in the maintenance of pluripotency has been recently established (Fu et al., 2018). Specifically, it has been demonstrated that two circRNAs, namely circBIRC6 and circCORO1C, are functionally connected with the maintenance of pluripotency in human embryonic stem cells and, in particular, circBIRC6 acts as a sponge for miR-34a and miR-145, relieving the suppression of NANOG, OCT4, and SOX2 espression (Yu et al., 2017). In an widely used vertebrate model, aimed at exploring circRNAs with potential functions during early vertebrate development, Liu et al. performed high-throughput sequencing, and applied the circRNA Identifier algorithm, throughout the duration of zebrafish embryo development (Liu et al., 2019); this study provided important information on the dynamic regulation of circRNAs implicated in the control of zebrafish differentiation and described novel specific circRNAs responsible for embryo development.

The possible role and impact of circRNAs in human development have been also recently reported with specific regard to cardiogenesis and neurogenesis (Lee et al., 2019). Of note, circRNAs were shown to be more abundant in the brain than in other tested organs in the adult mouse (You et al., 2015), pig (Veno et al., 2015), and human (Chen B. J. et al., 2019). The expression and roles of circRNAs in brain development and aging as well the implications in CNS diseases have been recently reviewed (Mehta et al., 2020).

## CircRNAs in Reproductive System and Germ Cell Development

During spermatogenesis and oogenesis, a tightly controlled expression of stage-specific genes is crucial for the normal development of gametes. Recently, circRNAs have emerged as a novel class of ncRNAs that regulate gene expression also in gametogenesis, but their role has not been completely clarified yet.

A study focused on the expression levels of circRNAs in brain, liver, heart, lung and testis, indicated that testis produces a huge amount of circRNAs, only second to that in brain (You et al., 2015), suggesting that circRNAs may have an important role in the testis function.

Notably, about 30 years ago the first circRNA was discovered in mouse as a transcript originating from the testis-determining gene Sry (Capel et al., 1993). In male, spermatogonial stem cells (SSCs) undergo self-renewal to ensure at the same time the maintenance of the stem cells pool and the differentiation to spermatocytes and spermatids. SSCs can also dedifferentiate into embryonic stem (ES)-like cells to acquire pluripotency *in vitro* (Conrad et al., 2008) and they are able to be reprogrammed to transdifferentiate to cell lineages of other tissues and for this reason SSCs have relevant applications in treating male infertility (Chen et al., 2017). Distinct circRNA expression profiles in different types of male germ cells indicate an important role exerted by circRNAs in the control of self-renewal and differentiation processes of SSCs (Zhou et al., 2019). By using highthroughput sequencing, circRNAs expression profiles have been identified in mouse male and female germline stem cells: a total of 18822 circRNAs were described in the germline stem cells and 921 circRNAs were differentially expressed between the male and female germline stem cells, suggesting that circRNAs could confer sex-specific properties needed for differentiation into gametes between male and female stem cells in mouse (Li X. et al., 2017; Li et al., 2019).

**FIGURE 3:**
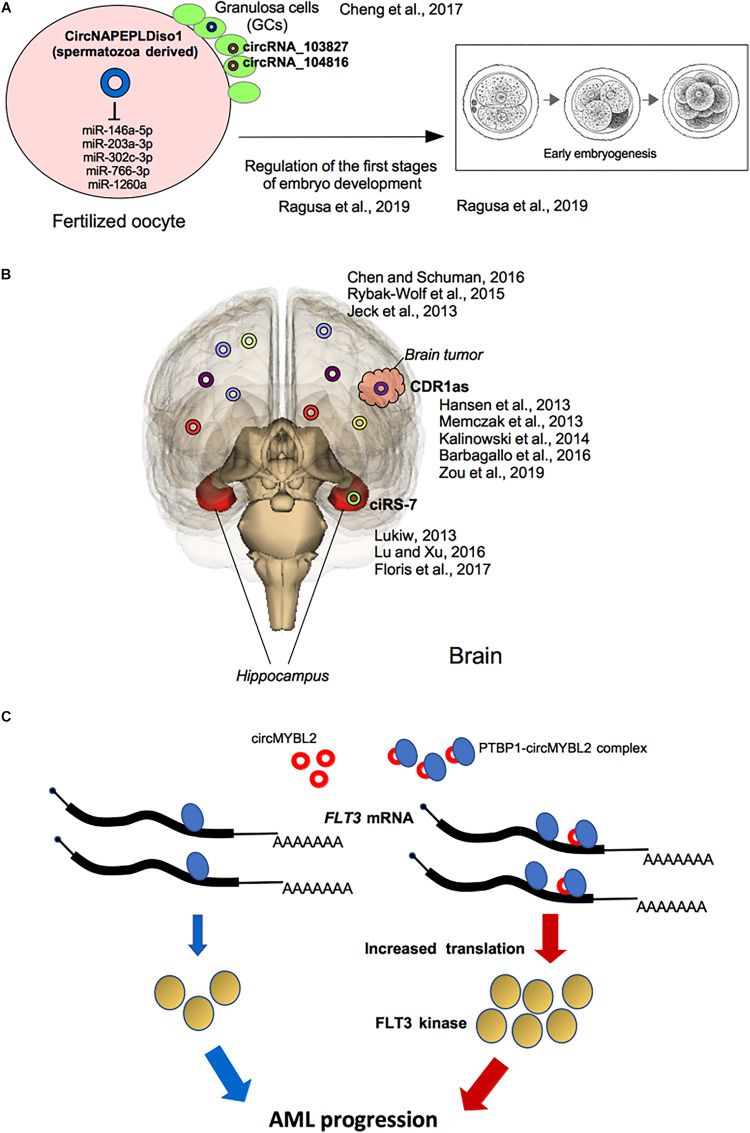
Selected functional effects of circRNAs in development and cancer. **(A)** Potential roles of circRNAs in reproduction: circRNAs expressed in granulosa cells (GCc) and in spermatozoa and involved in the first stages of embryo development into the fertilized oocytes are shown. **(B)** Roles of circRNAs in brain disease: in the Hippocampus, dysregulation of ciRS-7 expression is associated with Alzheimer’s disease and, generally, with neuronal-associated diseases. CiRS-7/CDR1as deregulated expression is also involved in brain tumorigenesis. **(C)** The PTBP1-circMYBL2 complex is highly expressed in AML patients with FLT3-ITD mutations where the translation of FLT3 mutated kinase is specifically induced fostering tumor progression.

Moreover, testis-derived circRNAs have been detected in human seminal plasma because they are resistant to exonuclease activity due to their circular form which confer them a great potential as liquid biopsy tools for various human diseases (Dong et al., 2016; Cai et al., 2018). Interestingly, in a recent study the expression of eight candidate circRNAs generated from six linear transcripts (CNR1, LEPR, MTHFR, NAPEPLD, NPC2, and SIRT1) has been profiled in five RNA samples from human and murine spermatozoa. Among them, authors focused on circNAPEPLDiso1, investigating its ability to bind miRNAs; they showed that circNAPEPLDiso1, expressed in mouse and human spermatozoa, specifically interacts with five miRNAs (miR-146a-5p, miR-203a-3p, miR-302c-3p, miR-766-3p, and miR-1260a) involved in the control of cell cycle and, some of them, expressed by the oocyte. This finding suggests a role of circNAPEPLDiso1 as a paternal-derived sponge for miRNAs inside the fertilized oocytes to regulate the first stages of embryo development by increasing levels of miRNA targets (Ragusa et al., 2019; Figure 3A).

An exhaustive review has recently described the potential roles of circRNAs in reproduction, particularly by analyzing circRNAs expression pattern in ovary (Quan and Li, 2018). Granulosa cells (GCs), the somatic cells surrounding oocyte, play an important role during oogenesis and early stages of embryo development (Moreno et al., 2015) and the study of circRNAs expressed in the GCs of subjects undergoing *in vitro* fertilization at a young age (less than 30 years) and at an older age (more than 38 years) showed that in older women, the expression of 46 circRNAs was up-regulated, whereas, 11 circRNAs were down-regulated. In particular, a negative correlation between the elevated expression of circRNA_103827 and circRNA_104816 in GCs and the top quality embryo number has been shown, suggesting that both circRNAs were closely related to decreasing ovarian reserve and adverse reproductive outcomes (Figure 3A). Therefore, circRNAs pattern of GCs may be used as potential biomarker to predict oocyte developmental capability and consequent assisted reproduction outcome (Cheng et al., 2017).

## CircRNAs in Cell Differentiation

Circular RNAs are expressed in several different organs following a spatial- and temporal-specific course, which suggests their potential biofunctions (Chen and Schuman, 2016; Zhao W. et al., 2019). To date, there is a growing number of studies reporting that circRNAs could be involved in the development of mammalian tissues as in neural development (van Rossum et al., 2016; Constantin, 2018), in osteogenic differentiation (Gu et al., 2017; Huang et al., 2019), in skeletal muscle development (Chen et al., 2020) or in hematopoiesis (Bonizzato et al., 2016).

### Neuronal CircRNAs

Several recent reports have shown that circRNAs are more enriched in neuronal tissues respect to other tissues (Chen and Schuman, 2016; Figure 3B). The reasons may be: (i) the brain has a consistent number of expressed circRNA host genes such as neuronal genes which regulate neurogenesis, neurodevelopment, and neuronal differentiation (Rybak-Wolf et al., 2015); (ii) neuronal genes contain very long introns (>10 kb), and circularized exons are more frequently flanked by long introns with inverted repeated sequences, thereby facilitating formation of circRNAs (Jeck et al., 2013); (iii) as circRNAs haven’t 5′ and 3′ ends, they are more stable than linear coding or non-coding RNAs, leading to a relatively longer half-life (Piwecka et al., 2017).

The first study of circRNAs in neuronal development documented their significant enrichment in brain and most of them were derived from host genes that code for synaptic proteins. The authors profiled the mouse circRNA population in the hippocampus over several stages: embryonic (E18), early postnatal (P1), postnatal at the beginning of synapse formation (P10) and late postnatal hippocampus following the establishment of mature neural circuits (P30) (You et al., 2015). They observed that circRNA expression pattern associated with the onset of synaptogenesis at P10. Interestingly, the circRNAs that were induced during hippocampal development were transcribed from the gene loci coding for proteins enriched with synapse-related functions (You et al., 2015). Interestingly, using high resolution *in situ* hybridization, for the first time, this study visualized circRNA punctae in the dendrites of neurons. These data show that circRNA expression levels are regulated by neural plasticity, suggesting their importance in regulating synaptic transmission and/or local translation.

As circRNAs are found to be preferentially expressed along neural genes and in neural tissues, several research groups focused their efforts on the study of the circRNAs involvement as new biomarkers for aging-correlated multiple sclerosis characterized by neurodegeneration, the mental illness schizophrenia and for the neurodegenerative pathologies as Alzheimer’s disease (AD), Parkinson’s disease (PD) (Ghosal et al., 2013; Lukiw, 2013). AD is the most common cause of dementia worldwide characterized by progressive dysmnesia, cognitive impairment, and psychiatric symptoms. Although the mechanisms of onset and progression of AD remain unknown, the primary clinicopathological characteristics of AD are aggregates of amyloid precursor protein-derived amyloid-β (Aβ) peptides and intraneuronal neurofibrillary tangles in the brain. Targeted molecular therapies for the treatment of AD have recently entered in medical practice (Chang et al., 2018).

Dysregulation of miR7-ciRS-7 interaction has been reported in the hippocampus of AD patients, where the expression of ciRS-7 is low and, therefore, the level of miR-7 is increased, with consequent down-regulation of miR-7 target mRNAs (Lukiw, 2013; Floris et al., 2017; Figure 3B). The dysregulation of the interaction between ciRS-7 and miR-7 has been reported to be crucial for other neuronal disorders, including PD, where ciRS-7 has a sponge activity on miR-7 expression (Lu and Xu, 2016; Floris et al., 2017; Figure 3B). In fact, miR-7 is highly expressed in cortical neuronal progenitors and its depletion causes microcephaly-like brain defects (Piwecka et al., 2017; Table 1).

**TABLE 1:** CircRNAs expressed in neuronal tissue and neuronal diseases.

circRNA	Function	References
ciRS-7		Hansen et al., 2013
		Floris et al., 2017
	Sponge activity on miR-7	Lu and Xu, 2016
		Sang et al., 2018
hsa_circRNA_104597	Valuable marker for schizophrenia	Yao et al., 2019
CDR1as		Hansen et al., 2013
		Kalinowski et al., 2014
		Uhr et al., 2018
	Sponge activity on miR-7	Tanaka et al., 2019
		Zhong et al., 2019
circFBXW7	Tumor suppressor	Yang et al., 2018
circSHPRH	Tumor suppressor	Zhang et al., 2018

Parkinson’s disease is characterized by loss of dopaminergic neurons in the substantia nigra, which leads to a series of motor function disorders including rest tremor, muscular rigidity, and bradykinesia. The overexpression and aggregation of α-synuclein (SNCA), which is present in Lewy bodies, is a distinctive diagnostic marker in PD (Rodriguez et al., 2015). It has been shown that miR-7 overexpression induced more efficient repression of SNCA in the empty HeLa cell line that did not express ciRS-7, suggesting that ciRS-7 may play a role in modulating SNCA through a miR-7-dependent pathway (Hansen et al., 2013). These results also suggested a possible sponge effect between ciRS-7 and miR-7 *in vitro*. Other studies reported that circSNCA can sponge miR-7 thereby up-regulating expression of SNCA mRNA, resulting in reduced autophagy and increased apoptosis in SH-SY5Y cells (Sang et al., 2018).

Schizophrenia (SZ) is a serious neuropsychiatric disorder with high recurrence and disability rates (van Os and Kapur, 2009). The pathogenesis of SZ is not yet fully understood and the most accredited causes seem to be environmental and genetic factors, although the lack of reliable biomarkers hinders early diagnosis and effective treatment of SZ patients (van Os and Kapur, 2009). Very recently, to assess whether expression of circRNAs in peripheral blood mononuclear cells (PBMCs) may be useful as low invasive biomarkers for diagnosis and/or therapeutic response in SZ patients, a research group analyzed circRNA expression profiles in PBMCs from SZ individuals and healthy controls (Yao et al., 2019). The expression of hsa_circRNA_104597 was assessed to be at low level in patients affected by schizophrenia (Yao et al., 2019; Table 1). ROC curve analysis showed that hsa_circRNA_104597 alone had a sensitivity of 84.31% and specificity of 86.41% respect to hormones (e.g., cortisol, insulin, leptin, prolactin, and growth hormone), miRNAs, lncRNA, indicating it as diagnostically valuable marker (Yao et al., 2019). In addition, they found that hsa_circRNA_104597 expression level increased after the treatment for 8 weeks with antipsychotic medications confirming it as potential therapeutic biomarker for SZ (Yao et al., 2019).

### CircRNAs in Osteogenic Differentiation

Bone remodeling is a dynamic process based on the balanced activities of the bone-forming osteoblasts (OBs), differentiating from bone marrow-derived mesenchymal stem cells (MSCs), and the bone-resorbing osteoclasts (OCs), multinucleated cells deriving from the monocyte/macrophage lineage (Berendsen and Olsen, 2015). Mature OBs produce and secrete proteins, such as alkaline phosphatase and type I collagen, which are necessary for the formation of the bone extracellular matrix, which then undergoes the process of mineralization. While most of the OBs die by apoptosis, some reach quiescence as bone lining cells on bone surfaces or become embedded in the bone matrix as osteocytes (Berendsen and Olsen, 2015). OCs control calcium and phosphate homeostasis, and play the role of mechano-sensors to respond to mechanical effort of the skeleton (Berendsen and Olsen, 2015). The cellular crosstalk of OBs and OCs is important to ensure bone integrity, repair, and calcium homeostasis, and imbalance between OB and OC activities can lead to bone diseases, such as osteoporosis and cancer-associated bone destruction (Berendsen and Olsen, 2015).

Several microRNAs and long non-coding RNAs have been reported as differentially expressed during osteogenesis (Ell and Kang, 2014; Huynh et al., 2017; Xie et al., 2017; Puppo et al., 2019). Their involvement in bone cancer and metastasis is well addressed (Puppo et al., 2019). Nevertheless, little is known about the regulation of the expression and the role of circRNAs in bone development and in bone pathologies.

Circular RNAs are emerging as important molecules that may regulate bone homeostasis. Using gene expression analysis, several circRNAs were found to be differentially expressed in MSCs undergoing OB differentiation, respect to their undifferentiated counterparts (Zhang et al., 2019). Some circRNAs were linked to miRNAs with osteogenic roles, indicating that these circRNAs potentially function in osteogenic differentiation of BMSCs (bone marrow stem cells) (Zhang et al., 2019). The authors identified a crosstalk between miR-199b-5p and circIGSF11 (Table 2). Silencing of circIGSF11 promoted osteoblast differentiation and increased the expression of miR-199b-5p (Zhang et al., 2019).

**TABLE 2:** CircRNAs in osteogenic differentiation and pathologies.

circRNA	Function	References
circIGSF11	Interaction with miR-199b-5p	Zhang M. et al., 2019
circ19142	Osteoblastic differentiation	Qian et al., 2017
circ5846		
hsa_circ_0006393	Sponge activity on miR-145-5p	Wang Y. et al., 2019
hsa-circ-0016347	Onco-circRNA in osteosarcoma	Jin et al., 2017
circHIPK3		Xiao-Long et al., 2018
circ_001569		
circ-Cdr1as	Tumor suppressor	
circ-Foxo3		

It has been reported that BMP2 promoted the proliferation of osteoblasts *in vitro* (Qian et al., 2017). RNA-seq analysis of BMP2-treated MC3T3-E1 cells has been performed to analyze differential expression of circRNAs during different osteoblast differentiation stages (Qian et al., 2017). 158 circRNAs were differentially expressed and, specifically, the expression of circRNA.5846, circRNA.19142 and circRNA.10042 was increased in the BMP2 treated group (Table 2). Circ.19142 and circ.5846 were found to be not only strongly associated with the positive regulation of developmental processes but also related to the fibroblast growth factor, epidermal growth factor, platelet-derived growth factor and Wnt signaling pathways, which are involved in cell growth and differentiation (Qian et al., 2017).

Recently, Wang X.B. et al. (2019) found that the overexpression of hsa_circ_0006393 increased the expression level of genes associated with osteogenesis (Table 2). Hsa_circ_0006393 is expressed mainly in the cytoplasm and nucleus of BMSCs. miR-145-5p was shown to be sponged by hsa_circ_0006393, thus increasing the expression levels of osteogenic genes during bone remodeling (Wang X.B. et al. (2019)).

Unfortunately, no functional analysis was carried out. Additional investigations and functional studies are required to address the biological role of circRNAs in bone differentiation and remodeling.

### CircRNAs in Skeletal Muscle Development

Recent studies reported that circRNAs are well expressed in skeletal muscle tissue, and their expression levels are regulated during muscle development and aging (Cai et al., 2019; Zhang M. et al., 2019). Skeletal muscle is the largest tissue in the body, playing an important role in locomotion and metabolism (Millay et al., 2013). Skeletal muscle has mature cells that are syncytial and can contain hundreds of nuclei; therefore, correct muscle growth and homeostasis are determinant for human mobility. Conversely, muscular diseases, such as muscular dystrophy, sarcopenia, atrophy, and cachexia, severely affect the everyday life of humans (Millay et al., 2013). Development and growth of muscle mainly rely on the proliferation and differentiation of myogenic stem cells. Most of the myogenic stem cells are derived from the mesodermal cell lineages (Kuang et al., 2007) and Pax3 and Pax7 paired box genes are the principal biomarkers for the myogenic stem cell (Buckingham and Relaix, 2007).

Regarding the expression of circRNAs in the muscle and in its development, many studies have been performed on mouse and chicken embryos (Chen et al., 2018; Shen et al., 2019).

Duchenne muscular dystrophy (DMD), which is characterized by a progressive decrease of muscle function, is caused by frame-shifting deletions or nonsense mutations in the DMD gene (Koenig et al., 1989), being this last among the first genes where circRNAs were identified as RNA circles consisting of exons that were skipped by alternative splicing (Surono et al., 1999). In contrast to the previous idea that circRNAs might be derived from aberrant splicing events (Sharp, 1994), the formation of circRNAs from the DMD gene is not necessarily created by the exon skipping, mainly because no strong correlation has found between the identified spliced transcripts and the circRNAs expected to be produced (Surono et al., 1999).

A recent work showed that most of the circRNAs expressed in myoblast during the growth stage regulated the cell cycle, while the circRNAs expressed in the differentiation stage are related with development activity (Zhang et al., 2018). For examples, circZNF609 showed higher expression in myotubes than in myoblasts and its downregulation reduced myoblast proliferation (Legnini et al., 2017; Table 3). In the mouse model a proposed mechanism implies that circZNF609 inhibits myoblasts differentiation by sponging miR-194-5p and upregulating BCLAF1 (Wang Y. et al., 2019).

**TABLE 3:** CircRNAs in skeletal muscle development and diseases.

circRNA	Function	References
circZNF609	Sponge activity on miR-194-5p	Legnini et al., 2017; Wang Y. et al., 2019
circLMO7	Sponge activity on miR-378a-3p	Miska et al., 1999; Wei et al., 2016
circSNX29	Sponge activity on miR-744	Peng et al., 2019
circLPAR1	Biomarker for the prognosis of MIBC	Lin et al., 2019

CircLMO7, derived from LMO7 gene, was highly expressed in skeletal muscle tissue. High levels of circLMO7 significantly decreased the expression of MyoD and myogenin (MyoG), suggesting that circLMO7 inhibited myoblast differentiation (Table 3). On the other side, circLMO7 overexpression increased myoblasts proliferation (Wei et al., 2016). From a mechanistic point of view, circLMO7 interacted with miR-378a-3p that targeted HDAC4 expression (Wei et al., 2016). CircLMO7 may serve as a decoy for miR-378a-3p, resulting in higher expression of HDAC4 thus decreasing the transcription of MEF2A and repressing myoblast differentiation (Miska et al., 1999).

Conversely, circSNX29 expression is correlated with myoblasts differentiation. It was reported that the expression level of circSNX29 was much higher in embryonic skeletal muscle than adult skeletal muscle and it was principally enriched in the cytoplasm (Peng et al., 2019; Table 3). CircSNX29 acts as a miR-744 sponge and increases Wnt5a and CaMKIId expression resulting in the activation of non-canonical Wnt pathways and myoblasts differentiation. Other groups have documented a similar “sponge” mechanism for other circRNAs, by sequestering miRNAs that regulate the expression of genes positively or negatively implicated in myoblasts differentiation (Li H. et al., 2018; Ouyang et al., 2018).

Many evidences of circRNAs dysregulation in muscle diseases are emerging. As previously mentioned, circRNAs produced by transcripts spliced from the DMD gene were among the first to be identified in skeletal muscles, mostly generated at the 5′ end of the transcript (Surono et al., 1999). Recently, a region spanning exon 45 to exon 55 of the DMD gene that represents a deletion hotspot in 63% of DMD patients was characterized (Suzuki et al., 2016). The authors reported that multiple exon skipping, targeting exon 45-55, was related with the increase of the symptoms in the patients identifying the favorite splice-sites involved in both circRNA formation and in multiple exon skipping of exon 45-55. The data confirm a circRNA-generation model in which the interaction between upstream and downstream introns triggers multiple exons skipping and creates circRNAs (Suzuki et al., 2016).

### CircRNAs in Hematopoiesis

Next Generation Sequencing (NGS) recently showed a conspicuous circRNA expression in human hematopoietic progenitors, and in differentiated lymphoid and myeloid cells (Nicolet et al., 2018). In the hematopoietic compartment, circRNAs are significantly enriched and secreted in vesicles named exosomes derived from platelets, where circRNAs resulted to be more abundant and widely expressed compared with other cell types (Preußer et al., 2018).

In the hematopoietic system, circRNA expression is cell-type specific, and it increases upon cell maturation. CircRNA splicing variants can also be cell-type specific (Nicolet et al., 2018). In the bone marrow, the hematopoietic stem cells (HSCs) differentiate into various progenitor cells, which in turn generate many types of myeloid and lymphoid cells requiring a tight regulation of gene expression of transcription factors and non-coding RNAs (Goode et al., 2016).

In a cutting-edge article dating back 20 years, Caldas et al. (1998) documented in the hematopoietic tissue the expression of circRNA isoforms of key genes such as MLL although the work was not very successful at the time, probably because the expression of circular isoforms was lower than mRNAs encoding the key transcriptional regulators.

## CircRNAs in Cancer: Novel Diagnostic and Therapeutic Biomarkers

One of the peculiar characteristics of circRNAs is that they are more resistant to the enzymatic activity of RNase R than linear mRNA, bypassing common RNA turnover steps (Kristensen et al., 2019). This feature has been exploited by liquid biopsies in the context of clinical trials conducted on various pathologies including cancer. Thanks to the stability of their structure in the longtime, it has been easy to trace the circRNAs in human tissues and fluids as serum and urine (Zhang et al., 2018). Their presence or absence in the fluids is emerging as an attractive diagnostic and prognostic tool and for this reason, in the field of translational medicine, they are becoming potent non-invasive biomarkers (Meng et al., 2017; Zhang et al., 2018).

The list of circRNAs involved in carcinogenesis continues to grow, however, the functional relevance of the vast majority is yet to be discovered. Some circRNAs can act as oncogenes and sustain proliferative signaling in cancer progression, while others may behave as tumor suppressors (Kristensen et al., 2018). Therefore, the cancer-specific expression status and functional mode of circRNAs may be used in cancer diagnosis and precise treatment in the future.

### CircRNAs in Hematological Disease

Hematological cancers are characterized by the aberrant growth of oligoclones of hematopoietic cells, which are able to invade the bone marrow and the blood, leading to severe anemia and immunodeficiency (Handschuh, 2019). Recently, using NGS technology, Salzman et al. (2012) addressed new cancer-specific fusion transcripts in hyperdiploid B-lineage acute lymphoblastic leukemia (ALL). They sequenced several RNA transcripts with numerous combinations in which the exons could merge (“scrambled exons”) and identified circularized RNAs. In five samples of hyperdiploid B-cell precursor-acute lymphoblastic leukemia they observed hundreds of circRNA transcripts with >700 circular isoforms where more than 10% of all transcripts derived by a comparable number of genes (Salzman et al., 2012). This study showed that many genes could produce scrambled exons (*ESYT2*, *FBXW4*, *CAMSAP1*, *KIAA0368*, *CLNS1A*, *FAM120A*, *MAP3K1*, *ZKSCAN1*, *MANBA*, *ZBTB46*, *NUP54*, *RARS*, and *MGA*) but they were expressed both in normal and blood cancer cells, not providing a more specific and useful interpretation of circRNA relevance for hematopoietic cell functions and pathology (Salzman et al., 2012). In a more recent study, RNA-seq analysis from whole-blood samples reported a very high number of expressed circRNAs, comparable to the brain (Memczak et al., 2015). Also in this case the functional aspect of circRNAs expression was not investigated but it was observed that hundreds of circRNAs were much higher expressed than corresponding linear mRNAs (Memczak et al., 2015).

If little is documented about the function of circRNAs in the development of hematopoietic tissue, much has been documented on their role in leukemias (Mei et al., 2019). Acute myeloid leukaemia (AML) represents the clonal expansion of hematopoietic precursors blocked at different stages of differentiation. The pathogenesis of AMLs is correlated to the presence of genetic alterations and the transcription factors regulating myelopoiesis are consistently involved in chromosomal translocation (Tenen et al., 1997). In AML the molecular event related to the transfation ability of chromosomal translocation-generated AML fusion proteins (such as PML/RARa, PLZF/RARa, AML1-ETO, MLL/AF9) is strictly dependent on their capacity to induce abnormal epigenetic modification on genes relevant to the transformation process (Grignani et al., 1998; Di Croce et al., 2002; Fazi et al., 2007). Of note, recently, fusion-circRNAs (f-circRNAs) derived from transcribed exons of chimeric proteins as MLL/AF9 and PML-RARA, generated by leukemia-associated chromosomal translocation, were discovered and demonstrated to be oncogenic by *in vitro* and *in vivo* experiments (Guarnerio et al., 2016; Table 4). Guarnerio et al. (2016) showed that *f-circPR* and *f-circM9* expression in leukemic cells increases cell proliferation and clonogenicity and that f-circRNA silencing reverted the phenotype, demonstrating that these f-circRNAs are biologically active and play pro-proliferative and pro-oncogenic activities. Recently it was reported that circ-VIM expression level in *de novo* AML patients [non-Acute Promyelocytic Leukemia (APL) patients with normal karyotype] was significantly upregulated compared with that in healthy controls (Yi et al., 2019; Table 4). Vimentin (VIM) is a component of type III intermediate filament protein, involved in the regulation of lymphocyte adhesion and transcellular migration, and is associated with poor clinical outcome in older patients with AML (Wu S. et al., 2018). Collectively, these results make circ-VIM as a promising diagnostic biomarker and treatment target in AML (Yi et al., 2019).

**TABLE 4:** CircRNAs in hematopoiesis and cancer.

circRNA	Function	References
f-circPR f-circM9	Pro-oncogenic activities in leukemia	Guarnerio et al., 2016
circ-VIM	Up-regulated in AML	Yi et al., 2019
circ-HIPK2	Biomarker in APL	Li et al., 2018
circMYBL2	Required for FLT3-dependent leukemia progression	Sun et al., 2019
circ-PVT1	Pro-oncogenic activities in AML	Hu et al., 2018
hsa_circ_0004277	Biomarker in AML	Li et al., 2018
circ-BA9.3	Pro-oncogenic activities in CML	Kaleem et al., 2015; Pan et al., 2018

Mutations within the FMS-like tyrosine kinase-3 (FLT3) gene, resulting in the internal tandem duplication (ITD; FLT3-ITD) or in the tyrosine kinase domain mutation (TKD; FLT3-TKD), occur in approximately 30% of AML patients. Recently, it has been shown that circMYBL2, a circRNA generated from the circularization of the cell-cycle checkpoint gene MYBL2, is crucial for FLT3-dependent leukemia progression. Mechanistically circMYBL2, by interacting with the polypyrimidine tract-binding protein 1 (PTBP1) to FLT3 messenger RNA significantly increases the protein level of mutant FLT3 kinase contributing to the AML progression. The depletion of this circRNA significantly impairs tumorigenicity of FLT3-ITD AML cells, highlighting this circRNA as putative relevant therapeutic target in this AML subtype (Sun et al., 2019; Figure 3C).

Of note, APL is a subtype of AML, characterized by the presence of the promyelocytic leukemia-retinoic acid receptor α (PML/RARα) fusion protein, which induces an oncogenic transcriptional silencing of the Retinoic Acid (RA) signaling pathway and causes the block of differentiation at the promyelocytic stage and neoplastic transformation of APL blasts (Grignani et al., 1998; Di Croce et al., 2002). Of note, the treatment of APL blasts with pharmacological doses of RA can overcome this repression and induce terminal differentiation *in vitro* and *in vivo* (Fazi et al., 2005). Gene expression analysis by RNA-seq showed 4,313 APL-expressed circRNAs in NB4 cells (Li et al., 2018). Furthermore, 508 circRNAs were expressed during all-*trans* retinoic acid treatment. The expression of circ-HIPK2 was lower in AML cells compared with APL cells, and overexpression of circ-HIPK2 increased differentiation in NB4 cells (APL cells with PML-RARA) (Li et al., 2018). Furthermore circ-HIPK2 had lower expression in APL samples of patients respect to that in healthy control samples and other subtypes of AML cases. The expression level of circ-HIPK2 significantly increased when APL patients achieved complete remission. This may suggest that circ-HIPK2 could act as a biomarker in APL cells.

Other circRNAs have been identified in hematopoietic malignancies as for example: circ-PVT1 in AML and in head and neck squamous cell carcinoma, where its expression is significantly associated with mutant p53 (Verduci et al., 2017; Hu et al., 2018; Table 4); hsa_circ_0004277, significantly lower in the AML than in healthy controls and patients who entered complete remission after treatment (Li W. et al., 2017; Table 4); circ-BA9.3 in chronic myeloid leukemia (CML), which is a stem cell disorder of uncontrolled myeloid proliferation characterized by the reciprocal translocation t(9;22) (q34; q11.2) resulting in the BCR-ABL1 fusion (Kaleem et al., 2015; Pan et al., 2018; Table 4).

Different cirRNA expression profiles correlate with different types of leukemia and clinical features, including tumor stage and recurrence, supported by recent RNA-seq studies. From all these studies it is clear that circRNA dysregulation signatures in cancers (in tissue- and development stage-specific manner), their tumor suppressive/oncogenic roles and stability and abundance in body fluids make them attractive non-invasive biomarkers in liquid biopsies.

### CircRNAs in Brain Tumors

Many articles about the involvement of circRNAs in brain tumors are emerging due to their versatility and hypothetical use for liquid biopsy-based diagnosis and prognosis. Glioma is a common type of central nervous system tumor where diffuse glioma (glioma cells exhibiting extensive invasive growth into the surrounding central nervous system) is the most frequent tumor especially in adults (Wesseling and Capper, 2018). Song et al. (2016) conducted a study that included seven oligodendrogliomas, 20 glioblastomas and 19 normal brain specimens to explore the expression level of circRNAs using high-throughput sequencing. To analyze the great number of raw data, the authors developed a sofisticated computational pipeline named UROBORUS. They found that the total number of detected circRNAs in GBM was significantly lower than that in normal brain tissue showing that eight highly expressed GBM-specific circRNAs might be good GBM-specific biomarker candidates (Song et al., 2016).

Among the first circRNAs identified and studied in brain cancer there is the one originating from the antisense transcript of the cerebellar degeneration-related protein 1 gene (CDR1as, also known as ciRS-7, previously described) that many reports have considered to have miRNA sponge activity (Hansen et al., 2013; Memczak et al., 2013; Kalinowski et al., 2014; Barbagallo et al., 2016; Zou et al., 2019; Table 1 and Figure 3B).

Mature ciRS-7 as is principally expressed in the cytoplasm, it has 74 miR-7 binding sites that can specifically bind to miR-7 molecules. As a result, the miR-7 target mRNAs are released from binding to miR-7 (Hansen et al., 2013; Kalinowski et al., 2014). The same seizure mechanism of ciRS-7 as toward miR-7 has been reported in development of nasopharyngeal carcinoma (Zhong et al., 2019), in colon cancer (Tanaka et al., 2019), in breast cancer (Uhr et al., 2018) and others.

Interestingly, Pamudurti et al. published that some circRNAs are associated with ribosomes suggesting a possible translation into proteins (Pamudurti et al., 2017). About this, it has been reported that circFBXW7 has coding potential and that translated peptide could be bound by an antibody targeted to related sequences holding potential prognostic implications in brain cancer (Yang et al., 2018). CircFBXW7 expression can significantly inhibit cell progression, migration, and tumor formation *in vivo* acting as tumor suppressor (Yang et al., 2018; Table 1). In this study FBXW7-185aa reduced the half-life of c-Myc by antagonizing USP28-induced c-Myc stabilization. An *in situ* GBM mouse model revealed the tumor suppressing effect of FBXW7-185aa but not of circ-FBXW7 circRNA with an IRES mutation. Also circSHPRH has coding abilities and leads to the formation of a peptide (Zhang M. et al., 2018). SHPRH-146aa is downregulated in GBM compared with para-cancerous tissues and overexpression of SHPRH-146aa significantly inhibited glioma growth in xenograft mouse models (Zhang M. et al., 2018; Table 1).

Altogether these data highlighted that circRNAs in brain tumors could be excellent biomarkers for their diagnosis, prognosis and classification. According to this idea, some groups reported that the knockdown of oncogenic circRNAs might be a reasonable approach for the treatment of glioma in the future. On the contrary, circRNAs that play tumor suppressor activity might be used in overexpression therapies. Novel approaches to express proteins acting as potent tumor suppressors in brain tumor models by engineering a circRNA vector have been also recently published, suggesting a circRNA-based treatment of glioma in the near future (Meganck et al., 2018; Wesselhoeft et al., 2018).

### CircRNAs in Osteosarcoma

Osteosarcoma (OS), a primary bone tumor arising from mesenchymal cells, has the highest fatality rate of all cancers among children and adolescents and many patients suffer from disease recurrence due to existing or potential distant metastasis (Ritter and Bielack, 2010). Patients with OS may benefit novel non-conventional therapies, such as small molecule-targeted drugs, but these strategies often lead to severe side effects and have failed in clinical trials (Otoukesh et al., 2018), therefore therapies focusing on complex gene regulation axes or networks are urgently needed. An increasing number of circRNAs that act as tumor suppressor or onco-circRNAs deregulated in bone cancer have been published. Below we describe the most significant studies in terms of inclusion of exploitation of patients’ cohorts and *in vitro* models.

Circular RNA hsa-circ-0016347 that derives from the *KCNH1* oncogene was found to have high expression levels in osteosarcoma tissue samples and in cell lines and to promote proliferation, invasion and metastasis *in vitro* and *in vivo* (Jin et al., 2017; Table 2). Zhang et al. (2017) found a circRNA from *UBAP2* gene to be the most upregulated circRNA in osteosarcoma patient samples respect to adjacent non-tumoral tissues. High expression of circUBAP2 correlated with lower overall survival of the patients, promoted osteosarcoma growth and inhibited apoptosis both *in vitro* and *in vivo*.

In OS cell lines, tissues and plasma circHIPK3 was demonstrated to be down-regulated together with circ_001569, ciRS-7 and circ-Foxo3 (Xiao-Long et al., 2018; Table 2). Moreover, the authors showed that patients with lower expression of circHIPK3 had shorter overall survival time than those with higher circHIPK3 expression. Furthermore, they showed that circHIPK3 expression was associated with several clinicopathological features of patients with OS (Xiao-Long et al., 2018; Table 2). CircHIPK3 levels were associated to Enneking stage and lung metastasis other than age, gender and tumor location according to statistical analysis. These results showed that lung metastasis and advanced cancer were associated with lower expression levels of circHIPK3 and suggested that circHIPK3 may be used as a biomarker for diagnosis and prognosis prediction of osteosarcoma.

### CircRNAs in Muscle-Invasive Cancers

Bladder cancer is the most common malignancy of the urinary system and it can be classified into two types according to the depth of cancer invasion: non-muscle invasive tumor (70–80%) and muscle-invasive tumor (20–30%). Patients with muscle-invasive bladder cancer (MIBC) present high incidence of metastasis and poor prognosis. Therefore, for MIBC patients it is urgent to find biomarkers for early diagnosis and to follow the progression of the disease during active surveillance by liquid biopsy (de Kruijff et al., 2020). Very recently a novel circRNA, circ lysophosphatidic acid receptor 1 (LPAR1) (hsa_circ_0087960), derived from two exons of 226 base pairs in length, has been identified in MIBC tissues (Table 3). circLPAR1 was found to be expressed at low level in a cohort of 125 cases of MIBC tissues. Furthermore, it predicted a worse disease-specific survival time than patients with high circLPAR1 expression (Lin et al., 2019). The authors showed the circLPAR1 may function as a potential novel biomarker for the prognosis of MIBC and may be associated with invasion and metastasis (Lin et al., 2019).

For an overview of the various circRNAs that have been found expressed in the various solid tumors as bladder cancer, breast cancer, colorectal carcinoma, esophageal squamous cell carcinoma, hepatocellular carcinoma, gastric cancer, lung and ovarian cancer, skin cancer, see the comprehensive review by Kristensen et al. (2018).

## CircRNAs in Cancer Stem Cells

cancer stem cells are a small proportion of cells considered the driving force of tumor initiation, progression, chemoresistance, relapse, and are also responsible for metastatic dissemination and therapeutic failure. By definition, both CSCs and normal tissue stem cells possess self-renewal capacity, however, in CSCs self-renewal is typically deregulated. Several lines of evidence have suggested that circRNAs might contribute to the stemness of cancer and to the resistance of cancer cells to chemotherapy. Recently, many studies have demonstrated that circRNAs may be stably expressed and present in relatively high quantities in human body fluids, such as saliva, plasma, serum and exosomes, which also makes circRNAs ideal candidates as non-invasive liquid biopsy biomarkers for cancer (Su et al., 2019).

Increasing evidence obtained analyzing circRNA/miRNA network by bioinformatics approaches has established that specific circRNAs are implicated in stemness via serving as miRNA sponges in different cancers, such as breast cancer (Yan et al., 2017), laryngeal cancer (Wu Y. et al., 2018), gastric cancer (Zhao X. et al., 2020), and hepatocellular carcinoma (Zhu et al., 2019). As for glioma, since circPTN was shown to sponge miR-145-5p, which is a negative regulator of stemness (Zhao X. et al., 2020) and self-renewal (Rani et al., 2013) and since circPTN expression was more than 10-fold higher in glioma cells compared with normal glial cells, Chen J. et al. (2019) hypothesized that circPTN may be a positive regulator of stemness. As expected, tumor sphere formation assays determined that circPTN promoted increased levels of stemness markers, such as Nestin, CD133, SOX2, and SOX9, and tumor growth *in vitro* and *in vivo*, primarily via sponging miR-145-5p/miR-330-5p (Chen J. et al., 2019).

Interestingly, a recent study showed that exosomes from CD133+ cells carrying circ-ABCC1 mediate cell stemness and metastasis in colorectal cancer, revealing that circ-ABCC1 may be used as a biomarker in CRC studies (Zhao H. et al., 2020).

In addition, the stem transcription factors upregulated by specific circRNAs can acts as positive regulators of circRNAs themselves, as revealed for SNAIL. It is a key transcription factor regulating many processes in tumor biology, such as the epithelial mesenchymal transition (EMT) and the induction and regulation of cancer stem cells. SNAIL is regulated by signaling networks involving plenty of ncRNAs, including circRNAs, which usually act as sponges for miRNAs targeting SNAIL. For example, in hepatocellular carcinoma (HCC), circ-ZNF652 is significantly upregulated and linked to highly metastatic features and poor prognosis, it physically interacts with miR-203 and miR-502-5p as a sponge to increase the expression of SNAIL. Interestingly, Snail, in turn, may also regulate circ-ZNF652 through physically binding to the E-box motif on the promoter of circ-ZNF652 to increase its expression. This loop thus forms a positive feedback that perpetuates the circ-ZNF652/miR-203/502-5p/Snail signaling axis (Guo et al., 2019; Skrzypek and Majka, 2020).

## Conclusion

Circular RNAs are emerging as an extremely relevant class of endogenous RNAs expressed abundantly by the transcriptome. They are characterized by a covalently closed loop structure, resulting in RNA molecules that are more stable than linear RNAs. Thanks to these molecular structural features, they are suitable to be considered excellent biomarkers for the diagnosis and prognosis in cancer disease using liquid biopsy techniques.

High-throughput sequencing technologies are revealing an increasing number of circRNAs in all human organs and systems, the deregulation of which is a very important source of knowledge because it can be exploited for the early diagnosis and/or for the prediction of the outcome in some tumors. The implementation of specific bioinformatic tools makes possible to predict the cellular functions of circRNAs in physiological conditions and in diseases.

The circRNAs-microRNA code, in particular, is emerging to have great impact on the regulation of gene expression during development and differentiation, as well as in diverse pathologies and cancer. The molecular stability of circRNAs provides a great potential which renders them good tools for therapeutic innovative strategies. Engineered circRNA delivery to cells via exosomes could be among the most important issues in the next years to improve the precision therapy against several diseases, including cancer.

## Author Contributions

All authors listed have contributed to collecting the data and to writing, improving, and finalizing the manuscript.

## Conflict of Interest

The authors declare that the research was conducted in the absence of any commercial or financial relationships that could be construed as a potential conflict of interest.

## Abbreviations

AD: Alzheimer’s disease
ALL: acute lymphoblastic leukemia
AML: acute myeloid leukaemia
APL: acute promyelocytic leukemia
BMSCs: bone marrow stem cells
circRNA: Circular RNA
CML: chronic myeloid leukemia
CSCs: cancer stem cells
DMD: Duchenne muscular dystrophy
EMT: epithelial mesenchymal transition
FLT3: FMS-like tyrosine kinase-3
GBM: glioblastoma multiforme
GCs: granulosa cells
HCC: hepatocellular carcinoma
HSCs: hematopoietic stem cells
MIBC: muscle invasive bladder cancer
miRNA: micro RNA
NGS: Next Generation Sequencing
OBs: osteoblasts
OCs: osteoclasts
OS: osteosarcoma
PD: Parkinson’s disease
PML/RAR α: promyelocytic leukemia-retinoic acid receptor α
RA: Retinoic Acid
RBP: RNA-binding protein
RNA-seq: RNA sequencing
SSC: spermatogonial stem cells
SZ: Schizophrenia
VIM: vimentin.
